# Interferon-λ Genetic Variations and Hepatitis C: Yet to be Discovered

**DOI:** 10.5812/hepatmon.19433

**Published:** 2014-05-10

**Authors:** Maryam Keshvari, Heidar Sharafi

**Affiliations:** 1 Blood Transfusion Research Center, High Institute for Research and Education in Transfusion Medicine, Tehran, IR Iran; 2 Middle East Liver Diseases (MELD) Center, Tehran, IR Iran

**Keywords:** Hepatitis C, Genetic polymorphism

***Dear Editor,***

The association of genetic factors with treatment induced hepatitis C clearance in Pakistani patients was recently demonstrated by Tipu et al. (1). They evaluated the impact of different genetic variants in interferon-λ (IFNL) genomic region on treatment induced hepatitis C clearance. The study was remarkable regarding the investigation of several numbers of single nucleotide polymorphisms (SNPs) in IFNL region. We would like to express our comments regarding the study. We observed that the patients in the study were treated with interferon (IFN) alpha 2b and ribavirin (RBV). Since the combination of pegylated IFN and RBV has been approved for treatment of chronic hepatitis C from 2002, the conventional regimen (IFN alpha 2b and RBV) for treatment of hepatitis C virus (HCV) infected patients is a debate point. Also, we have a concern regarding two terms through the article, first the authors defined the term sustained virologic response (SVR) as undetectable HCV RNA at the end of treatment course, whereas SVR must be defined as undetectable HCV RNA 24 weeks after treatment completion; second the term spontaneous clearance which refers to clearance of HCV from serum without treatment was misused through the article. Interestingly, the current study showed significant association between different IFNL polymorphisms and treatment response in HCV genotype 3 infected patients in our neighbor country (Pakistan). The results of previous studies searching for the association of IL28B SNPs and SVR in patients infected with HCV genotype 2/3 were conflicting and most of them did not find any association between IL28B SNPs and SVR (2, 3). Also, in this study, 50 SNPs were genotyped of which 25 of them were included in the final association analysis and 13 of them were found to be associated with the treatment response (1). The role of SNPs in the IFNL region remained mysterious and yet to be discovered. There are two main hypotheses for the role of these SNPs:

1. Combined effect of SNPs which refers to independent impact of each SNP;
2. Causal and Tag SNPs which is defined by the linkage disequilibrium (LD) between few tag SNPs and a causal SNP (sometimes undiscovered or untested).

Most of the SNPs in IFNL region including the SNPs found by Tipu et al. (1) to be associated with treatment response are in linkage disequilibrium (LD) which favors the Causal and Tag SNPs hypothesis. On the other hand, Prokunina-Olsson et al. (4) discovered a new IFNL gene which was assigned as IFNL4 and its encoded protein was observed to possess antiviral activity. Also, in the latter study, a genetic variant called ss469415590 (TT/ΔG) found to be located in exon one of IFNL4 gene which its TT allele does not express the protein and was associated with favorable treatment outcome and its ΔG allele expresses the IFNL4 protein and was associated with treatment nonresponse. The results of most previous studies looked for the functional role of IFNL SNPs showed the pretreatment expression of interferon stimulated genes (ISG) to be lower in patients with SVR when compared to patients with treatment failure. The same finding was observed for the expression of ISGs and IL28B genotypes in which the expression of ISGs were lower in patients with IFNL favorable genotypes than in patients with IFNL unfavorable genotypes (5, 6). However, the results of studies investigated the association of IL28B genotypes with IL28B expression were inconsistent which makes it difficult to draw the final role map (5-9). Discovery of IFNL4 gene and the exon one TT/ΔG SNP was a clue for solving the puzzle of IFNL genetic variations and HCV treatment response. If we accept the ss469415590 as the casual variant, the association of the IFNL genetic variants, IFNL4 expression, the subsequent ISGs expression, and the ultimate differences in treatment response seems to be relevant. In conclusion, studies such the one by Tipu et al. can open new horizons in diagnosis, management, and evaluation of hepatitis C prognosis in near future.
